# Low-dose N-acetyl cysteine prevents paraquat-induced mortality in *Caenorhabditis elegans*

**DOI:** 10.17912/micropub.biology.000815

**Published:** 2023-03-23

**Authors:** Candelaria Gonzales-Moreno, Lucia E Fernandez-Hubeid, Andrea Holgado, Miriam B Virgolini

**Affiliations:** 1 Departamento de Farmacología Otto Orsingher. Facultad de Ciencias Químicas. Universidad Nacional de Córdoba, Córdoba, Argentina; 2 IFEC-CONICET. Departamento de Farmacología Otto Orsingher. Facultad de Ciencias Químicas. Universidad Nacional de Córdoba, Córdoba, Argentina; 3 Department of Biological Sciences, St. Edward's University, Austin, TX, USA

## Abstract

Exposure to the herbicide paraquat (PQ; 1,1′-dimethyl-4,4′-bipyridinium dichloride) affects the redox balance of the cell, an effect that can be restored by antioxidants, including N-acetyl cysteine (NAC). One hour of exposure to PQ (0 mM, 10 mM, 50 mM, or 100 mM) dose-dependently increased mortality in *Caenorhabditis elegans* after exposure (immediate toxicity), while this effect was more evident 24 hours thereafter (delayed toxicity). Importantly, pretreatment with NAC 0.5 mM for one hour partially prevented mortality in the immediate assay, while it had no effect in the delayed test, revealing the importance of long-term studies when evaluating toxicity.

**Figure 1. Survival expressed as a percent of lethality:**
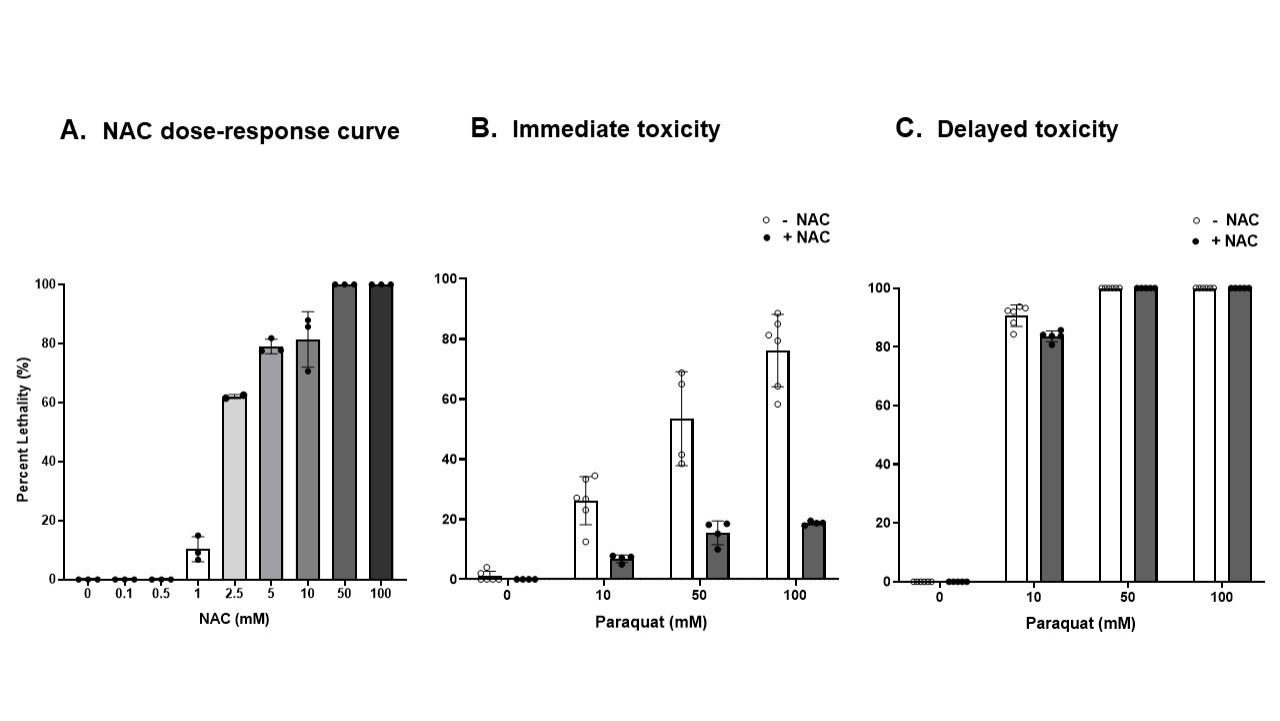
**A.** **Effect of one-hour NAC exposure on L4- worms' survival.** *p<0.05; ***p<0.001 respect to the control group. **B.** **Lethality measured after one-hour PQ exposure preceded by one-hour 0.5 mM NAC treatment (immediate toxicity assay).** **p<0.01; ***p<0.001 with respect to the control group, and ^##^ p<0.01 and ^###^ p<0.001 related to the respective PQ-exposure concentration. **C.** **Lethality measured 24 hours after one-hour PQ exposure preceded by one-hour 0.5 mM NAC treatment (delayed toxicity assay).** ***p<0.001 with respect to the control group, and ^###^ p<0.001 related to the respective PQ-exposure concentration. The dots indicate the number of assays averaged from tests performed in triplicate. The bars show the percentage of dead animals + the standard error.

## Description

**Introduction**

The invertebrate *Caenorhabditis elegans* ( *C.* *elegans* ) is a powerful and versatile biological model to assess the adverse effects of many toxicants, representing an intermediate approach between *in vitro* assays and mammal studies (Hunt, 2017; Meyer and Williams, 2014) .

Paraquat (PQ; 1,1'-dimethyl-4-4'-bipyridyl dichloride) is a toxic, widely-used, non-selective herbicide. The oxidative damage to living organisms is the result of the action of the NADPH cytochrome P-450 reductase and mitochondrial complex I by catalyzing the formation of a mono-cationic free radical, which is rapidly re-oxidized to superoxide in the presence of oxygen (Dinis Oliveira et al., 2006; Thirugnanam and Santhakumar, 2022) . In this process, glutathione (GSH) levels are depleted and glutathione disulfide (oxidized glutathione, GSSG) increases, a reaction that occurs particularly in the dopaminergic neurons, providing the basis of the association between PQ and the etiology of parkinsonism (Bastias Candia et al., 2019). In this line, *C. elegans* has become a useful model for the study of PQ-induced toxicity and for investigating the mechanisms underlying potential therapeutic drugs because it has a GSH cycle similar to that of mammals, allows genetic manipulation (Maulik et al., 2017) , and accurately reproduces mitochondrial dysfunction (Ferguson et al., 2019; Dilberger et al., 2019) .

The potent antioxidant N-acetyl cysteine (NAC), a reactive oxygen species scavenger, is considered a precursor of GSH biosynthesis (Tardiolo et al., 2018) that along with the conjugation with electrophiles and metal chelation constitutes the main cytoprotective mechanism (Mlejnek, 2022) . In this regard, recent evidence suggests that these beneficial effects are mediated by the sulfur species resultant of NAC metabolism, which may have a direct role in the elimination of oxidants and the enhancement of cellular reduction capacity (Brandan Pedre et al., 2021). Moreover, experimental evidence in mammals indicates that NAC can increase life expectancy in wild-type or complex I-deficient strains (Polyak et al., 2018) , as well as induce resistance to environmental stressors such as PQ (Oh et al., 2015; Desjardins et al., 2017) .

Based on these observations, this study aimed to assess the lethality of the prooxidant environment induced by PQ and to evaluate whether NAC mitigates this toxicity with potential therapeutic implications in pathologies associated with redox imbalance and mitochondrial dysfunction.

**Results**

The results obtained are presented in Figure 1 (A-C). To determine the concentration of NAC to be used as a possible mitigator of PQ toxicity, NAC alone was tested to see if it affected *C.* *elegans* viability (Fig 1. A). The observed differences in worm survival demonstrate the complexity of the drug's biological properties. In effect, both 0.1- and 0.5-mM NAC showed no lethality one hour after exposure. However, a survival dose-dependent increase in lethality was observed between the 2.5- and 10-mM NAC concentrations that reached a maximum at 50- and 100-mM NAC: F (8,18) = 479.41, p<0.0001. Thus, based on the toxicological parameters, 0.5 mM NAC was the maximal concentration at which there is a N o-Observed- A dverse- E ffect L evel (NOAEL) and was thereby selected for the following experiments.

Lethality measured 1 h after PQ exposure increased in a dose-dependent fashion following the herbicide concentration, with 100 mM PQ inducing almost 80% of lethality ( Fig. 1 . B) **.** However, one-hour pre-treatment with 0.5 mM NAC followed by PQ at different doses (10, 50, and 100 mM) prevented and significantly reduced mortality at all three doses when the animals were observed immediately thereafter (immediate toxicity). A two-way ANOVA revealed a significant effect of PQ: F (3,26) = 43.11, p<0.0001; NAC: F (1,26) = 91.41, p<0.0001 and the overall interaction: F (3,26) = 15.25, p<0.0001.

**Table:** 

By comparison, the delayed assay demonstrated that the percentage of worms that survived dramatically decreased 24 hours after PQ exposure ( Fig. 1 . C). In effect, a 90% mortality was observed at the lower PQ concentration (10 mM) that reached maximal lethality (100%) at the two higher doses evaluated (50- and 100-mM PQ). Provided the high incidence of mortality, in this case, the pre-treatment with 0.5 mM NAC for one hour was able to partially reverse this effect, a fact that is evident in the two-way ANOVA results that yielded the following effects: PQ: F (3,28) = 2784.32, p<0.0001; NAC: F (1,28) = 6.69, p<0.05, and the overall interaction: F (3,28) = 10.93 p<0.0001.

**Discussion**

The evidence presented here indicates that, as expected, low millimolar NAC concentrations (0.1-1 mM) did not affect *C. elegans’* survival. However, higher NAC concentrations (2.5 mM and above) increased mortality in a dose-dependent fashion until maximal lethality was obtained at both, 50- and 100-mM NAC. These findings explain why the 0.5 mM NAC dose was selected to mitigate PQ toxicity in our experiments. Coincidentally, prolonged treatment with 5- or 15-mM NAC to young or aged *C. elegans* dose-dependently reduced *C. elegans* ’ survival and lifespan (Gusarov et al., 2021) . However, in other reports, 10 mM did not affect life length and survival (Yang and Hekimi, 2010) , while long-term exposure to 1-, 2-, or 5-mM NAC has been described to increase the same parameters, revealing that prolonged exposure to low NAC doses may cause enduring protective effects in the worms (Oh et al., 2015) . This evidence contrasts with the one-hour exposure protocol to 2.5- and 5-mM NAC doses employed in the present study that resulted in high mortality. The differences between both studies may lie in the length of NAC exposure (dietary in Oh et al., 2015 vs. a short exposure, in this study).

Regarding evidence in other invertebrates, Shaposhnikov et al., 2018 evaluated NAC effects in the nM, µM, and mM concentration range on *Drosophila melanogaster* survival. The results indicate that low NAC doses increased the lifespan in flies continuously exposed to the antioxidant, while doses of 10 mM or above decreased survival in males. In females, on the contrary, all NAC concentrations significantly reduced survival (Shaposhnikov et al., 2018) . A similar effect was reported in *Drosophila melanogaster* , with 60 m NAC increasing the lifespan, while higher doses (120 mM NAC) reduced the flies’ lifespan (Brack et al.,1997). To investigate possible mechanisms for the action of NAC, various groups have used cell culture and posit that the expression of enzymes that control the formation and conversion of reactive oxidative stress through apoptotic and apoptotic-like features are essential (Liu et al., 2017) (Mlejnek et al., 2021) . More recently, Hwang et al., 2022 demonstrated that 0.1-10 mM NAC can cause oxidative, apoptotic, and excitotoxic neuronal death in mouse neuronal cultures that did not extend to the glial tissue.

Regarding the effects of PQ alone, the results reported by us demonstrate that this herbicide dose-dependently increased mortality in *C. elegans* , an effect that is further evident in the delayed toxicity study. Moreover, we determined that the lethal concentration 50 (LC50), was around the 50 mM PQ concentration when the animals were observed immediately after exposure to the herbicide for one hour. Similar to our work, a recent report shows that PQ (25- mM-250 mM) dose and time-dependently increased *C. elegans* mortality (Xie et al., 2020) . In the same line, Ji et al (2022) informed that PQ exposure (80 mM for 3 hours ) was able to kill all worms when observed immediately after that (Ji et al., 2022) . In contrast to our experiments, these worms were aged on FURD-added NGM/OP50 plates, which can constitute a confounding factor due to its intrinsic toxicity.

About the beneficial effects of NAC on PQ survival rates, our results demonstrated that NAC markedly reduced *C. elegans* mortality assessed immediately after one hour of PQ exposure, promoting survival of up to 80% of the population (vs. a 20% survival after PQ alone). This ameliorative effect was less prominent in the delayed test, where it was observed that 50 mM and 100 mM PQ induced lethality in 100% of the population, an index that NAC did not mitigate. Therefore, it is clear that PQ exposure affects *C. elegans* lifespan, and NAC can reverse that effect mainly when short-term toxicity is evaluated. This is probably the result of NAC properties acting as a GSH precursor restoring normal ROS levels against the oxidative microenvironment created by PQ (Dinis Oliveira et al., 2006) . In this regard, Oh et al, 2015 demonstrated that 24-hour exposure to 1-, 5-, or 10-mM NAC increased worms’ survival in response to 20 mM PQ, while 50 mM NAC, on the contrary, markedly increased the worms’ mortality. In comparison, our data demonstrated that a small NAC concentration and a short exposure could mitigate PQ-induced immediate toxicity. Other reports indicate that mM NAC concentrations reduced *D. melanogaster* survival in both sexes in the presence of 20 mM PQ, starting at 24 hours post-NAC exposure (Shaposhnikov et al., 2018) .

In addition, it is worth mentioning that sublethal PQ concentrations have been used in the PQ-induced Parkinson’s disease (PD) model in *C. elegans* (Bastias Candia et al., 2019; Dinis Oliveira et al., 2006). In this respect, Dilberger et al., 2019 using 5 mM PQ, described the association among oxidative stress, restricted energy metabolism, reduced stress resistance, and longevity, all keynote features of parkinsonism. Chronic PQ exposure (0.2-1.6 mM for 3-6 days) to L2 larvae dose-dependently decreases survival, induced motor deficits, and elicited dopaminergic degeneration all associated with parkinsonism (Wu et al., 2018) . Moreover, exposure to low doses and short times (0.5 mM PQ for 30 min) decreased population survival by 50% in L1 *C. elegans* larvae evaluated 24 hours after exposure. In this case, pre-or-post exposure to a natural antioxidant ( *Butia eriospatha* , 5-500 µg/ml) failed to prevent PQ-pro-oxidant consequences on survival (Tambara et al., 2020) .

Overall, this study has implications in the field of PD, a chronic and progressively debilitating pathology, in which the 90% of cases are ascribed to environmental factors, including the herbicide PQ (Maulik et al., 2017; Chia et al., 2020) . Thus, studies such as the present represent an advance not only in the knowledge of the mechanistic basis of neurological diseases but also provide evidence of the importance of timing in toxicity assessment.

## Methods

**Methods**

Approximately 20-30 well-fed worms belonging to the N2 Bristol strain in the L4 stage were exposed to 0 mM (control), 10 mM, 50 mM, or 100 mM PQ (Methyl viologen dichloride hydrate, Sigma Aldrich, Argentina) for one hour in a liquid medium (sterile water, total volume of 500 µL) with continuous shaking at 20 ^o^ C. The samples were run in triplicate and repeated 3-6 times in different worm populations. After one hour (immediate assay) or 24 hours (delayed assay), lethality was evaluated in seeded agar plates divided into four quadrants. A worm was considered dead when there was no movement after a gentle touch with the platinum loop. The exposure time was selected based on preliminary results where worms were exposed to 10 mM PQ for 24 hours resulting in a 100% mortality.

Optimal NAC (Pura Química, Argentina) concentration was obtained from a dose-response curve performed with 0.1 mM, 0.5 mM, 1 mM, 2.5 mM, 5 mM, 10 mM, 50 mM, and 100 mM NAC. The samples containing approximately 20-30 worms were run in triplicate with a final volume of 500 µL, incubated for one hour with continuous shaking at 20 ^o^ C, and transferred after that to a plate with seeded agar to count the dead and live worms under the microscope.

Based on the obtained results and in identical experimental conditions, approximately 20-30 worms in the L4 stage were pre-exposed to 0.5 mM NAC for one hour. Subsequently, PQ (0, 10, 50, and 100 mM) was added and incubated for an additional hour. After exposure, lethality was determined immediately (one hour after PQ exposure) or 24 hours thereafter to evaluate the impact of NAC on PQ’s immediate and delayed lethal effects.

The data were analyzed with one-way or two-way ANOVAs, as corresponds followed by a Tuckey’s test as a *post hoc* .

## Reagents

N2 strains were obtained from the Caenorhabditis Genetics Center (CGC), University of Minnesota, NM, USA, which is funded by NIH Office of Research Infrastructure Programs (P40 OD010440)

PQ (Methyl viologen dichloride hydrate) was purchased from Sigma Aldrich, Argentina. 

NAC was purchased from Pura Química, Argentina.

NGM medium; concentrated E. coli OP50 bacteria and all other reagents were prepared with analytical-grade products.
